# Real-Life Feasibility of HIV Drug Resistance Testing Using Dried Filter Analytes in Kenyan Children and Adolescents Living with HIV

**DOI:** 10.1128/spectrum.02675-21

**Published:** 2022-04-07

**Authors:** Akarsh Manne, Alexander DeLong, Winstone Nyandiko, Allison K. DeLong, Rachel Vreeman, Vladimir Novitsky, Anthony Ngeresa, Edwin Sang, Ashley Chory, Josephine Aluoch, Eslyne Jepkemboi, Millicent Orido, Celestine Ashimosi, Festus Sang, Joseph W. Hogan, Rami Kantor, for the RESPECT (Resistance in a Pediatric Cohort) Study

**Affiliations:** a Brown University, Providence, Rhode Island, USA; b Academic Model Providing Access to Healthcare (AMPATH), Eldoret, Kenya; c Moi University College of Health Sciences, Eldoret, Kenya; d Icahn School of Medicine at Mount Sinai, New York, New York, USA; Kumamoto University

**Keywords:** HIV, drug resistance, resource limited settings, hemaspots, dried blood spots, dried filter analytes, Kenya, youth with HIV

## Abstract

HIV-1 drug resistance remains a global challenge, yet access to testing is limited, particularly in resource-limited settings. We examined feasibility and limitations of genotyping using dried filter analytes in treatment-experienced Kenyan youth with HIV. Youth infected with HIV perinatally were enrolled in 2016–2018 at the Academic Model Providing Access to Healthcare in Eldoret, western Kenya. Samples were shipped in real-time at ambient temperature to the US, and those with viral load (VL)>1,000 copies/mL were tested based on convenience. Dried blood spots genotyping was attempted when unsuccessful from Hemaspots. Multiple logistic regression was used to examine predictors of genotyping success. Samples from 49 participants (median age 15 years, 43% female, median CD4 496 cells/μL [18%], median 8 years on therapy, median VL 11,827 copies/mL) were shipped after median 7 days from collection, arrived in 20 shipments after median 5 days, and extracted after median 2 days (1 day for samples processed on arrival; and 42 days for frozen Hemaspots). Overall, 29/49 (59%) samples with VL > 1,000 copies/mL and 25/32 (78%) with VL > 5,000 copies/mL were genotyped by either Hemaspots or DBS. Successful genotyping was associated with higher Hemaspot volume and higher VL. Real-life HIV-1 drug resistance testing from dried filter analytes is feasible, even in settings with constrained resources. Findings, particularly relevant where resistance testing is limited for clinical care, raise awareness to implementation practicability of this guidelines-recommended test in care of more individuals and populations. Further optimization of filter analytes is needed to overcome related challenges.

**IMPORTANCE** In this manuscript we use dried filter analytes shipped from Kenya to the US in real time, to demonstrate the real-life feasibility of conducting HIV drug resistance testing in a vulnerable population of young children and adolescents with HIV in a resource limited setting. Such testing, which is recommended in resource-rich settings, is unavailable in most resource limited settings for individual clinical care. We show that real-life HIV drug resistance testing from dried filter analytes is feasible, even in settings with constrained resources. These findings raise awareness to the importance of HIV drug resistance for individual care, even in such settings, and emphasize the implementation practicability of this guidelines-recommended test.

## INTRODUCTION

HIV drug resistance (DR) challenges sustainable antiretroviral therapy (ART) (1, 2). Treatment failure and DR are common in perinatally infected children and adolescents with HIV (CAWH), due to limited ART options and adherence challenges (3). The gold standard for DR testing is plasma, due to high accuracy and yield (2). However, plasma requires stringent storage conditions to maintain sample integrity, limiting feasibility and affordability (4).

Dried filter analytes (DFAs) like dried blood spots (DBS) can be stored and shipped at ambient temperatures, are considered plasma-equivalent, and are used for DR surveillance in resource limited settings (RLS) (5–11). Limited data exist on their use for clinical settings. Hemaspots, a newer DFA, hold larger volume (up to 200 μL), and importantly, combine an absorbent paper and desiccant within a plastic cartridge, preventing the need for separate drying (12, 13).

To address limited DR testing access in RLS, our objective was to establish the proof-of-concept feasibility for the primary outcome of successfully obtaining a sequence from any DFA in treatment-experienced Kenyan CAWH. Accomplishing this process in settings with limited infrastructure could support using this sequence for DR interpretation and improving the care of this and other vulnerable populations.

## RESULTS

**FIG 1:**
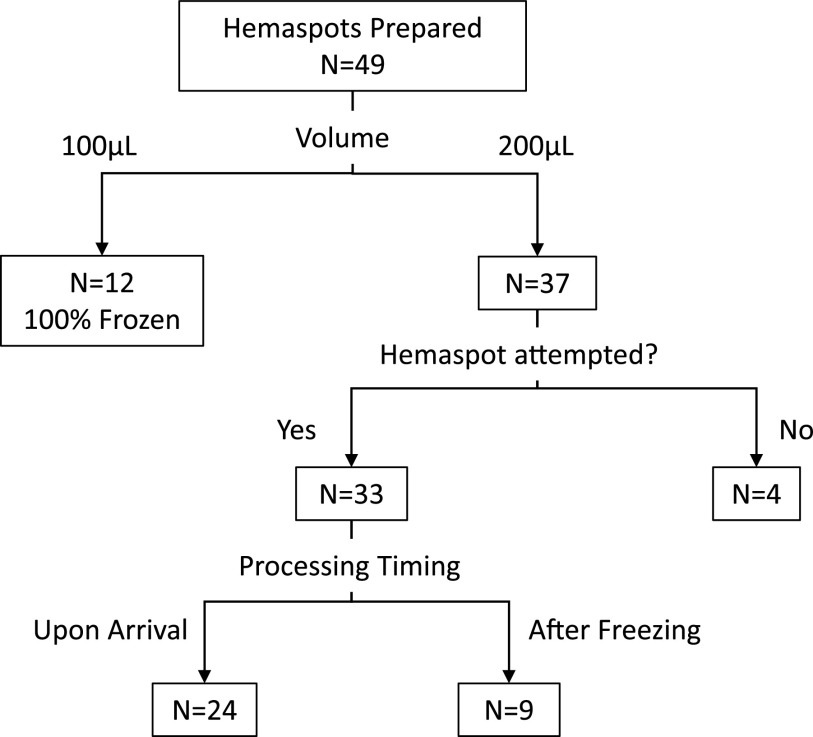
This flow diagram describes the flow and characteristics of the shipped samples according to sample preparation, and Hemaspot volume, genotype attempt and processing timing. All DBS were frozen and all failed Hemaspot genotyping was attempted from DBS.

Of 65 participants with VL > 1,000cmL who had Hemaspot and/or DBS prepared, 49 had both analytes, and were shipped. In those 49, median age was 15 years, 43% were female, with median CD4 496 cells/μL (18%), and median 8 years on ART. The median VL was 11,827cmL and 32/49 (65%) had VL > 5,000cmL. Fig. 1 and Table S1 demonstrate flow and characteristics of the 20 shipments (49 samples), of which six (21 samples) were immediately frozen.

Overall, 29/49 (59%) samples with VL > 1,000cmL and 25/32 (78%) with VL > 5,000cmL were successfully genotyped from any DFA (Tables S1, S2; Fig. S1). Genotypes were obtained from 14/45 (31%) Hemaspots, all 14 with VL > 5,000cmL (14/32 with VL > 5,000cmL, 44%). The 12 100 μL Hemaspots had median VL 2,432cmL, including only five with VL > 5,000cmL. The 37 200 μL Hemaspots had median VL 16,844cmL, including 20/24 handled immediately with VL > 5,000cmL, and 7/9 frozen with VL >5,000cmL (Table S2). A larger (nonsignificant) percentage of frozen Hemaspots were successfully genotyped versus those processed immediately (56% versus 38%; Fisher exact *P*-value = 0.422). Similarly, among 27 samples with VL > 5,000cmL, 5/7 (71%) frozen and 9/20 (45%) processed immediately were successfully genotyped (Fisher exact *P*-value = 0.385).

The 35 participants for whom Hemaspot genotyping was unsuccessful or not attempted had median VL 5,291cmL, 18/35 with >5,000cmL. Overall, 15/35 (43%), and 11/18 (61%) with VL > 5,000cmL were successfully genotyped from DBS.

Genotyping from 100 μL Hemaspots (all frozen) was less successful than from 200 μL Hemaspots (0/12 versus 14/33, Fisher exact *P* = 0.009), as well as from those prepared with 200 μL Hemaspots that were frozen (0/12 versus 5/9, Fisher Exact *P* = 0.04). In multiple logistic regression analysis, obtaining a genotype from either DFA was associated with higher VL. Hemaspot genotyping success was associated with higher VL and shorter time in transit. DBS genotyping success was also associated with higher VL (Table 1). See Supplemental Material for additional results.

**TABLE 1:** Genotyping success by analyte from multiple logistic regression analyses^*a*^^,^^*b*^

Covariate	200 μL hemaspot genotype (*n* = 32^*c*^)	DBS genotype (*n* = 34)	Hemaspot or DBS Genotype (*n* = 48)
Per 1 log10 VL unit higher	7.01 (1.48, 33.24) *P* = 0.014	5.72 (1.32, 24.71) *P* = 0.020	11.14 (2.60, 47.66) *P* = 0.001
Per day longer between collection and ship	0.92 (0.73, 1.15) *P* = 0.454	0.94 (0.80, 1.10) *P* = 0.410	0.90 (0.78, 1.05) *P* = 0.170
Per day longer between ship and receive	0.25 (0.06, 0.99) *P* = 0.049	0.92 (0.31, 2.79) *P* = 0.885	0.69 (0.26, 1.81) *P* = 0.447
Frozen versus processed upon arrival	1.64 (0.18, 14.84) *P* = 0.660	2.37 (0.28, 19.72) *P* = 0.425	1.48 (0.26, 8.33) *P* = 0.655

a One lost-then-found sample that was 22 days in transit was removed from analyses.

b All values are odds ratios (95% Confidence Intervals).

c Four samples that were not attempted by Hemaspot were removed from analyses. VL, viral load.

## DISCUSSION

Several informative observations can be made from this study. First, individualized DR testing in RLS is feasible and the median collection-to-extraction time of 14 days for nonfrozen samples is reasonable for clinical management even in developed settings, comparing favorably to an attempt in South African adults using whole blood and a laboratory-adjacent clinic (14). Although using DFAs creates additional challenges (as below) it improves feasibility for RLS, even without nearby capacity.

Second, using both DFAs, genotyping success of 59% (VL > 1,000cmL) or 78% (VL > 5,000cmL), though low, are within the range of reports using DBS in not fully controlled settings (8, 13, 15–17). The apparent improved success of DBS over Hemaspot (DBS yielded genotypes when Hemaspots failed) should be interpreted cautiously, since repeating Hemaspots genotyping, not performed here, could have been successful. Importantly, the circumvention of separate Hemaspot drying, mandatory for DBS, justifies their continued consideration, particularly where drying may be challenging.

Third, increased genotyping success was associated with higher VL, a known concept for DFAs (8, 9, 13, 18). This observation is further supported by the better genotyping success observed with high-volume Hemaspots. This VL limitation remains a challenge, even with the existing higher (VL > 1,000cmL) World Health Organization definition of treatment failure. Benefits from handling DFAs without refrigeration are countered by potential sample degradation in uncontrollable conditions and use of an in-house genotyping method (19).

Fourth, longer transit time was associated with less successful Hemaspot genotyping, but not DBS. This observation, extending reports in more controlled settings (11, 19), is likely related to increased nucleic-acid degradation with time, but perhaps also to larger Hemaspot volume and the ‘built-in’ desiccant resulting in insufficient drying, compared to DBS; a speculation that should be investigated. It does suggest potential benefit from shortening any step in sampling-to-extraction. An important way to reach this goal, beyond attempts to improve process logistics, is to build local DR testing capacity, as done by the World Health Organization (20).

Limitations of this study, which mandate interpretation caution, include small sample size, heterogenous parameters that could influence genotyping, no use of skin pricks, and lack of DFA validation. However, this study was not designed as validation, but rather proof-of-concept to demonstrate feasibility of real-life transcontinent DFA DR testing.

In summary, despite complexity and divergence in analytes (Hemaspots/DBS), storage temperature (frozen/not), Hemaspot volume (100/200 μL) and VLs, we demonstrate real-life feasibility of HIV DR testing from DFAs in RLS, which could be used to inform clinical care. Findings are particularly important for vulnerable populations like ART-experienced CAWH, as they mature into adulthood with accumulated DR (21). This feasibility should continue to raise awareness of the need to implement this guidelines-recommended test for more individuals. Optimization of low-cost, easy-to-use DFAs should continue, with focus on capacity building, technology transfer, alleviating logistics, and improvement of analytes.

## MATERIALS AND METHODS

Perinatally infected CAWH at the Academic Model Providing Access to Healthcare (AMPATH) in Eldoret, western Kenya were enrolled in 2016–2018 (22, 23). Blood was collected at four clinics: one central, adjacent to the laboratory; and three rural, average 40 miles away. Upon collection, blood was transferred within 4–6 h to the laboratory, where DFAs were prepared. DBS (Whatman 903; Fisher Scientific) were prepared by pipetting 50–75 μL blood onto each spot, drying overnight, and placing in a Bitran bag with humidity-indicating desiccants. Hemaspots were prepared by pipetting 100 or 200 μL blood on the device and closing the lid per manufacturers’ protocol (12). Both DFAs were at ambient temperature until courier-shipped at ambient temperature to the US, occurring every 2 weeks.

Upon arrival, if feasible considering time constraints, Hemaspots with VL > 1,000cmL were left at ambient temperature and attempted for genotyping within 1 day. If not feasible, samples were frozen at −80°C and attempted for genotyping when convenient. DBS were stored at −80°C and attempted if Hemaspot genotyping was unsuccessful. The last four samples with VL < 5,000cmL were genotyped from DBS only, since Hemaspot success for such samples was low.

Proportions and exact 95% confidence intervals (CIs) were calculated for three outcomes: Hemaspot genotype success, DBS genotype success among Hemaspot genotype failures, and genotype success by either DFA, defined as overall genotyping success.

Multiple logistic regression was used to examine predictors of genotyping success. Generalized additive models (GAMs) were used to examine relationships between continuous predictors and log odds of genotype success. Analyses were performed in R version 4.0.2 (*P*-value < 0.05 considered significant) (24). See Supplemental Material for additional methods.
