# Supplementary material for: Real-Life Feasibility of HIV Drug Resistance Testing Using Dried Filter Analytes in Kenyan Children and Adolescents Living with HIV
Source: Microbiol Spectr. 2022 Apr 7;10(2):e02675-21. doi: 10.1128/spectrum.02675-21 (PMC9045389; doi:10.1128/spectrum.02675-21)
Supplement: SUPPLEMENTAL FILE 1 — Supplemental material. Download Supplementary_File_1.pdf, PDF file, 0.1 MB [file supplementary_file_1.pdf]

**Supplemental Material**

Methods

Study participants were perinatally-infected CAWH who were enrolled in a prior parent study(1, 2). Demographic, clinical, and laboratory data were derived from AMPATH records(3). Samples were handled to minimize time between collection and nucleic acid extraction, while considering real-life constraints. CD4 and VL (Abbott M2000, Abbott Molecular, Inc., Des Plaines, IL) testing were performed at the AIDS Clinical Trials Group (ACTG)-accredited AMPATH Reference Laboratory.

For Hemaspot genotyping, membranes were removed from the device and placed into a 2 mL tube with 700µL of phosphate buffered saline (PBS). The membrane was incubated at room temperature for 30 minutes on a rotating shaker and total nucleic acid was extracted utilizing the EZ1 Advanced XL System (Qiagen; Hilden, Germany). Genotyping was performed using Sanger Sequencing by an in-house method as previously described(4). DBS genotyping was performed by cutting 3 spots and placing them into a 2mL tube with 700µL of PBS. Subsequent extraction and genotyping were similar to Hemaspots.

Participant characteristics were summarized, including age, gender, CD4 and ART. HIV-1 subtyping and DR interpretation were performed with Stanford Database tools(5). We hypothesized that Hemaspot genotyping success will be impacted by Hemaspot volume (100 vs 200uL), VL, and shipment logistics, including time lag between collection and shipment, shipping time, and freezing upon arrival. As needed, covariates were transformed ($log_{10}$ or square root) to ensure linearity and goodness of fit.

The study was approved by US (Lifespan; Indiana University; Mount Sinai) and Kenya (Moi University School of Medicine; Moi Teaching and Referral Hospital) ethics committees.

Results

Of 495 parent study participants, only 310 (63%) had Hemaspots and/or DBS prepared, due to delayed supplies availability. ART failure occurred in 21% of the whole cohort (105/495; published elsewhere(6)), and 21% of those with Hemaspots and/or DBS (65/310).

Upon arrival to the US, indicators for all samples in all shipments demonstrated adequate humidity.

Most (n=37/49, 76%) of the shipped Hemaspots were made with devices that held 200μL. All (12/12) 100μL Hemaspots and 24% (9/37) of the 200μL Hemaspots were frozen upon arrival. The number of viremic samples per shipment ranged from 0 to 7, depending on enrollment pace. There was a median seven (IQR = 3 to 13) days between sample collection and shipment, a median five (IQR = 4 to 6) days between shipment and arrival in the US, and a median two (IQR = 0 to 42) days between arrival and nucleic acid extraction. This differed for Hemaspots that were processed upon arrival or frozen: median 1 (IQR = 0 to 1) day for those processed upon arrival, and median 42 (IQR = 24 to 52) days for frozen Hemaspots. There was a median 41 (IQR = 12 to 73) days between arrival and extraction for attempted DBS. One sample, lost in the mail and subsequently retrieved, had a long, 22-day, shipment time and was omitted from the logistic regression analyses.

Of the 29 participants failing ART with available genotypes, 19 (66%) had HIV-1 subtype A, five (17%) subtype D, three (10%) subtype C and two (7%) recombinants (AC and AD). NNRTI-based regimens were taken by 19 (66%), and PI-based regimens by 10 (34%). DR was detected in 26/29 (90%), including 90% to NNRTI, 72% to NRTIs, and 72% dual class. None had PI resistance.

References

1. Vreeman RC, Nyandiko WM, Liu H, Tu W, Scanlon ML, Slaven JE, Ayaya SO, Inui TS. 2014. Measuring adherence to antiretroviral therapy in children and adolescents in western Kenya. J Int AIDS Soc 17:19227.
2. Vreeman RC, Nyandiko WM, Liu H, Tu W, Scanlon ML, Slaven JE, Ayaya SO, Inui TS. 2015. Comprehensive evaluation of caregiver-reported antiretroviral therapy adherence for HIV-infected children. AIDS Behav 19:626-34.
3. Tierney WM, Beck EJ, Gardner RM, Musick B, Shields M, Shiyonga NM, Spohr MH. 2006. Viewpoint: a pragmatic approach to constructing a minimum data set for care of patients with HIV in developing countries. J Am Med Inform Assoc 13:253-60.
4. Kantor R, DeLong A, Schreier L, Reitsma M, Kemboi E, Orido M, Obonge S, Boinett R, Rono M, Emonyi W, Brooks K, Coetzer M, Buziba N, Hogan J, Diero L. 2018. HIV-1 second-line failure and drug resistance at high-level and low-level viremia in Western Kenya. AIDS 32:2485-2496.
5. Shafer R. Stanford HIV Sequence Database. Available at http://hivdb.stanford.edu; acccessed September 10, 2021.
6. Nyandiko W, Holland S, Vreeman R, DeLong AK, Manne A, Novitsky V, Sang F, Ashimosi C, Ngeresa A, Chory A, Aluoch J, Orido M, Jepkemboi E, Sam SS, Caliendo AM, Ayaya S, Hogan JW, Kantor R, Resistance in a Pediatric Cohort S. 2022. HIV-1 Treatment Failure, Drug Resistance, and Clinical Outcomes in Perinatally Infected Children and Adolescents Failing First-Line Antiretroviral Therapy in Western Kenya. J Acquir Immune Defic Syndr 89:231-239.

**Table S1. Characteristics of 20 shipments of dried filter samples from Kenya to the US.**

| Batch | Number of Samples with VL >1,000 | Volume (µL) | Frozen (yes/no) | Range of Days from Collection to Hemaspot Extraction | Available Genotypes | VL [a] of Successes [b] | VL [a] of Failures |
|---|---|---|---|---|---|---|---|
| 1 | 0 | | - | - | - | - | - |
| 2 | 7 | 100 | Yes | 46-88 | 3/7 | 41200, 42300, 3000 | 9300, 1800,1300, 1200 |
| 3 | 5 | 100 | Yes | 67-72 | 2/5 | 64200, 25800 | 1900, 1500, 1000 |
| 4 | 3 | 200 | Yes | 48-54 | 2/3 | **22500**, 2517300 | 1100 |
| 5 | 3 | 200 | Yes | 28-33 | 3/3 | **19500**, **136800**, 16800 | - |
| 6 | 2 | 200 | Yes | 21-35 | 2/2 | **72800**, 2000 | - |
| 7 | 0 | 200 | - | - | - | - | - |
| 8 | 2 | 200 | No | 12 | 2/2 | **23000**, **25400** | - |
| 9 | 1 | 200 | Yes | 25 | 1/1 | **339200** | - |
| 10 | 2 | 200 | No | 11-18 | 1/2 | **90200** | 1500 |
| 11 | 1 | 200 | No | 23 | 1/1 | **11800** | - |
| 12 | 1 | 200 | No | 24 | 1/1 | 18200 | - |
| 13 | 2 | 200 | No | 10-13 | 1/2 | **11300** | 5300 |
| 14 | 1 | 200 | No | 9 | 1/1 | **469900** | |
| 15 | 1 | 200 | No | 15 | 1/1 | **111800** | |
| 16 | 2 | 200 | No | 12-29 | 0/2 | - | 2300, 63800 [d] |
| 17[c] | 4 | 200 | No | 13-16 | 4/4 | **111800** , 2200, 8000, 8200 | |
| 18[c] | 3 | 200 | No | 22 | 1/3 | 14700 | 3700, 1100 |
| 19 | 3 | 200 | No | 8-15 | 1/3 | 46400 | 3000, 2000 |
| 20[c] | 6 | 200 | No | 8-17 | 2/6 | **7500**, 1200 | 217600, 6800, 24500, 2900 |

Footnote: [a] viral load rounded to nearest 100 copies/mL. [b] Hemaspot successful genotypes in bold; DBS successful genotypes underlined; [c]Genotyping from Hemaspots in these shipments was attempted only for samples with VL>5,000 copies/mL; [d] sample was lost in the mail and retrieved after 22 days. Abbreviations: VL, viral load.

**Table S2. Genotyping success according to sample type, shipment time and viral load.**

| | Sampled to Shipped (Days[a]) | Shipped to Received (Days[a]) | Received to Extracted (Days[a]) | Genotype Success (Copies/mL) | |
|---|---|---|---|---|---|
| | | | | VL>1,000 | VL>5,000 |
| All (n=49) | 7 | 5 | 2 | 29/49 (59%) | 25/32 (78%) |
| HS 100µL (n=12; all frozen) | 17 | 4 | 52 | 0/12 (0%) | 0/5 (0%) |
| DBS (all frozen) | 17 | 4 | 94 | 5/12 (42%) | 4/5 (80%) |
| HS 200µL processed upon arrival (n=24) | 7 | 5 | 1 | 9/24 (38%) | 9/20 (45%) |
| DBS (all frozen) | 7 | 5 | 32 | 5/15 (33%) | 5/11 (45%) |
| HS 200µL frozen (n=9) | 3 | 5 | 20 | 5/9 (56%) | 5/7 (71%) |
| DBS (all frozen) | 3 | 5 | 58 | 3/4 (75%) | 2/2 (100%) |

Footnote: [a] Median. Abbreviations: DBS, dried blood spots; HS, Hemaspots; VL, viral load

**Figure S1. Genotyping success according to dried filter analyte and viral load.**

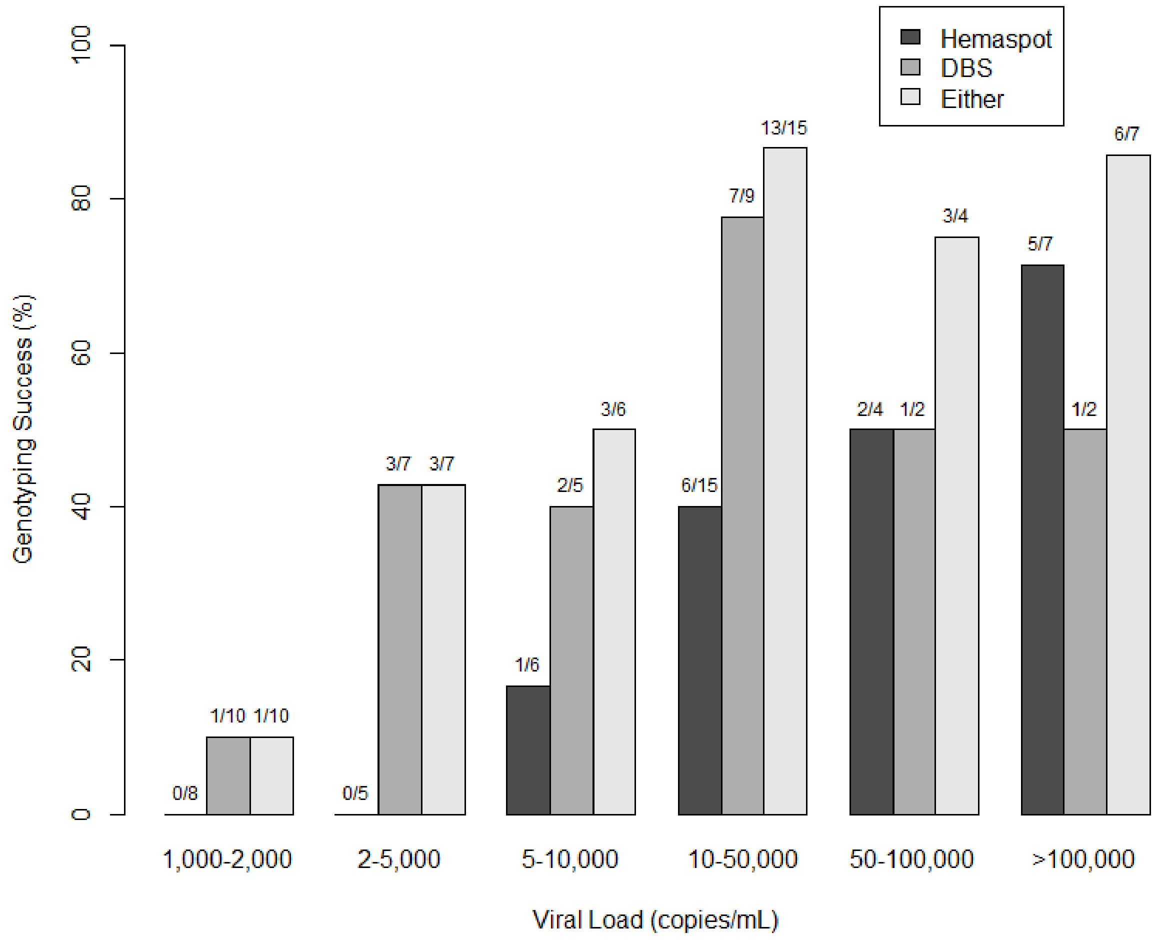


## Figure Legend

Figure S1

The graph depicts percentages of genotyping success (Y axis) from Hemaspots (n=45; black bars), DBS (n=35; dark gray bars), or either Hemaspot or DBS (n=49; light gray bars), by viral load (X axis). Shown above the bars are number of samples that were successfully amplified over the total number of samples that were attempted (n/N).
